# Computational study of effect of radiation induced crosslinking on the properties of flattened carbon nanotubes†

**DOI:** 10.1039/d2ra05550c

**Published:** 2022-10-11

**Authors:** Prashik S. Gaikwad, Malgorzata Kowalik, Adri van Duin, Gregory M. Odegard

**Affiliations:** Michigan Technological University Houghton MI 49931 USA gmodegar@mtu.edu; Pennsylvania State University, State College PA 16801 USA

## Abstract

Flattened carbon nanotubes (flCNTs) are a primary component of many carbon nanotube (CNT) yarn and sheet materials, which are promising reinforcements for the next generation of ultra-strong composites for aerospace applications. Significant improvements in the performance of CNT materials can be realized with improvements in the load transfer between flCNTs, which are generally oriented at different angles with respect to each other. An intriguing approach to improving the load transfer is *via* irradiation-induced chemical crosslinking between adjacent flCNTs. The objective of this research is to use molecular dynamics (MD) simulations to predict the behavior of flCNT junctions with 0- and 90-degree orientations and varying levels of crosslinking. The results indicate that crosslinking improves the flCNT interfacial load transfer for both orientations, but degrades the flCNT tensile response. The primary toughening mechanism at the flCNT/flCNT interface is the formation of carbon chains that provide load transfer up to the point of total rupture. Based on these results, it is clear that irradiation-induced crosslinking is beneficial in CNT-based composite systems in which interfacial load transfer between flCNTs is of primary importance, even though individual flCNTs may lose some mechanical integrity with crosslinking.

## Abstract 2

Molecular dynamics simulation of functionalized flattened carbon nanotubes.

## Graphical abstract

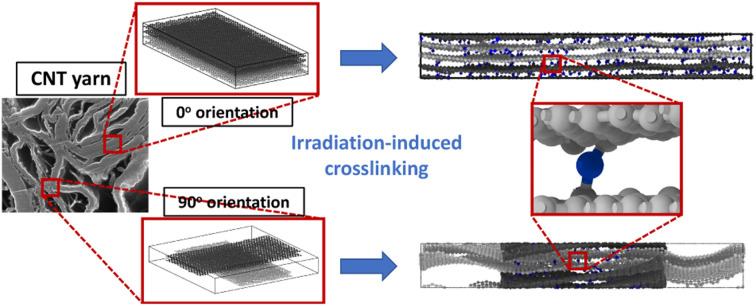

## 1 Introduction

For future deep space manned missions to be affordable and efficient, there is a need to build ultra-high-strength light-weight composite materials for structural components of aerospace vehicles. Carbon nanotube (CNT) materials, because of their exceptional specific strength and stiffness, are promising reinforcement candidates for this purpose.^1,2^ In particular, flattened CNTs (flCNTs) exhibit self-alignment and efficient packing in stacked arrays and can be used with high-performance resins to make the next generation of composite materials.^3,4^ However, TEM-observed fracture surfaces of flCNT materials have revealed intra-stack sliding in addition to complete stack pull-out when subjected to external loads.^3^ Since the CNT yarns suffer from poor internal load transfer,^5^ and flCNTs are major constituent of CNT yarns,^3^ a major challenge to utilize flCNTs in structural composites is achieving sufficient levels of flCNT/flCNT interfacial load transfer.

Several computational and experimental studies have shown that ion or electron beam (e-beam) irradiation is a feasible option to induce crosslink formation between adjacent CNTs for improved load transfer.^6–8^ Filleter *et al.* experimentally demonstrated up to 16-fold increases in the mechanical properties of double-walled CNTs following high-energy e-beam irradiation *via* TEM.^9^ Locascio *et al.*^10^ observed that irradiation-induced crosslinking improved the strength of multi-walled CNTs by more than a factor of 10 with a small reduction in the strain to failure. Post irradiation, Miller *et al.*^11^ observed a 48% increase in tensile strength of functionalized CNT sheets. Using the molecular dynamics (MD) approach, Pregler *et al.*^12^ studied the effect of ion and e-beam irradiation crosslinking on the failure of MWCNT inner-tube sliding and observed a significant increase in the force required for multi-walled CNT failure. Peng *et al.*^13^ experimentally demonstrated that e-beam irradiation leads to multi-shell failure in multi walled CNTs, which drastically improves the sustainable loads by a factor of 2–11 as compared to the non-irradiated samples. Kis *et al.*^14^ observed a 30-fold increase in the bending modulus of single walled CNTs exposed to e-beam treatment. Astrom *et al.*^15^ observed an increase in stiffness and strength of CNT materials with irradiation induced crosslinks. Using MD, Cornwell and Welch^16^ observed an increase in tensile strength of fibers (aligned CNTs) up to 60 GPa due to interstitial crosslinks. Despite the previous computational and experimental studies on the effect irradiation-induced crosslinks on CNTs, there is an insufficient understanding of how irradiation-induced crosslinks between flCNTs affects mechanical performance of flCNT arrays.

The objective of this research is to determine the effect of irradiation-induced crosslinking on the properties of flCNT composites using MD simulation. As the flCNTs within CNT yarns and bundles are entangled with each other at different orientations,^17^ two extreme orientation cases of flCNTs junctions were considered: 0° and 90°, which represent two aligned and perpendicular flCNTs, respectively. In this study, crosslinking is defined as the ratio of the number of carbon atoms forming the bonds between the two flCNT sheets to the total number of carbon atoms in the overlapped area. For each of the orientation cases, the amount of crosslinking was varied from 0% to 20%. For these models, the shear and transverse strengths were determined to identify the role of the flCNT alignment and irradiation-induced crosslinks on junction performance. The 0° orientation model was used to study the effect of crosslinking (and the associated flCNT wall damage) on the axial properties when the flCNT sheets are pulled along the armchair (along the *x*-axis) and zigzag (along the *y*-axis) directions. It is important to note that similar to Patil *et al.*,^18^ Pisani *et al.*,^19^ Deshpande *et al.*,^20^ and Gaikwad *et al.*,^21^ experimental validation of the predicted mechanical response is not performed because the relevant experimental characterization methods for this material have not yet been developed and performed, mostly because of the very small length scales involved.

## 2 Molecular modeling

The LAMMPS (Large scale Atomic/Molecular Massively Parallel Simulator) software package^22^ was used to perform the MD simulations in this study. The Reactive Force Field (ReaxFF) developed van Duin *et al.*^23^ was used to describe the interatomic forces using the C/H/O/N parameterization of Kowalik *et al.*^24^ ReaxFF is a bond-order force field, which directly enables bond formation and scission during MD simulations. For ReaxFF, different parameter sets are developed for specific applications. Thus, the accuracy of ReaxFF simulations is highly dependent on the choice of an appropriate parameter set. The parameter set used in this study was originally developed to investigate the chemical reaction processes during carbonization of oxidized polyacrylonitrile and poly(*p*-phenylene-2,6-benzobisoxazole), and incorporates parameters developed by Srinivasan *et al.*^25^ and Ashraf and van Duin.^26^ The performance and accuracy of this parameterization was demonstrated by Gaikwad *et al.*^21^ for systems containing flCNTs and amorphous carbon. The atomistic visualizations provided herein were created using the OVITO software package.^27^

### 2.1 flCNT sheets

**Fig. 1:**
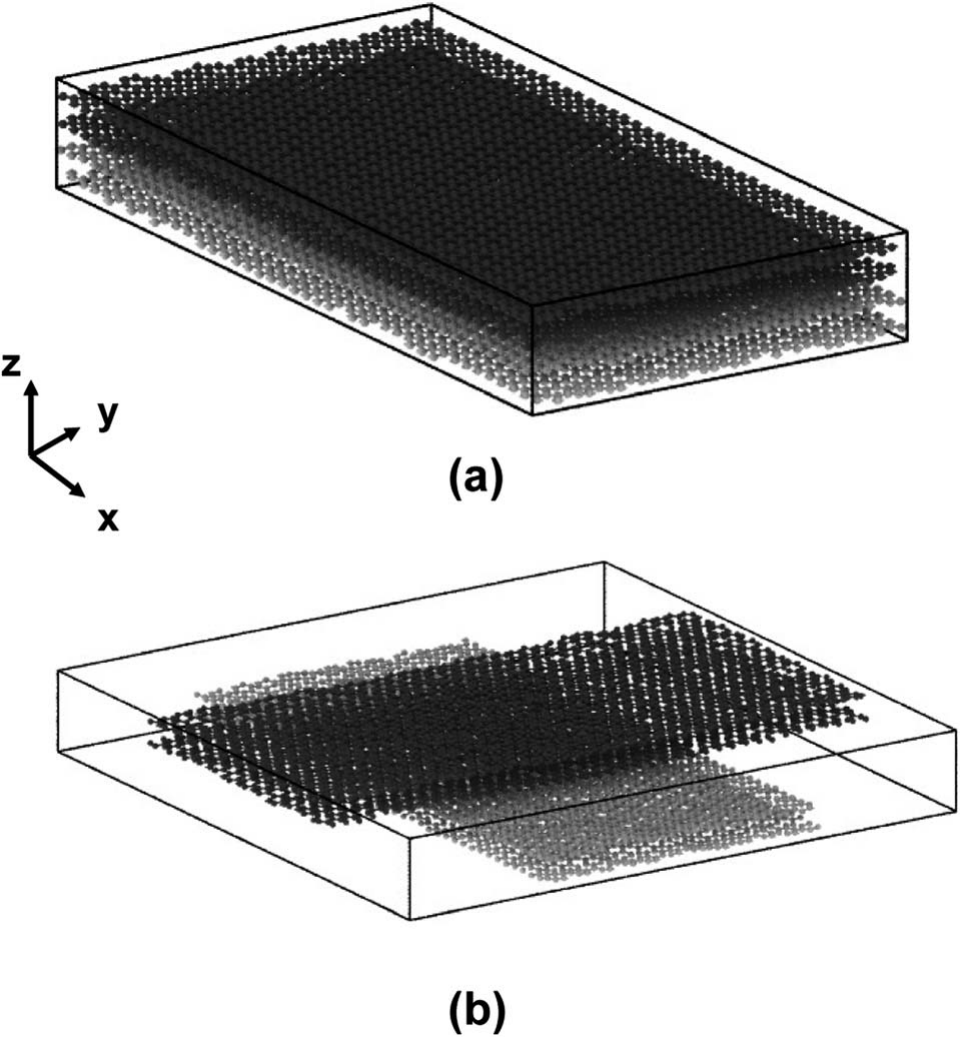
(a) 0° orientation model of two flCNTs and (b) 90° orientation model. The dark and light grey colors distinguish the two flCNT sheets.

Previous experimental and modelled studies have shown that flCNTs have distinctive flat regions and dumbbell-shaped end lobes.^3,28–31^ In this study, only the flat region is modelled, similar to Patil *et al.*,^18^ Pisani *et al.*,^19^ Deshpande *et al.*,^20^ and Gaikwad *et al.*^21^ The dumbbell-shaped end lobes are excluded for computational efficiency, while the interaction of these dumbbell shaped end lobes can be accurately captured with mesoscale modeling.^32–34^ In this study, each flCNT is modelled as two sets of graphene sheets representing the flat region of flCNTs. The “lattice” command in LAMMPS was used to create the models of the graphene sheets. The lattice parameters for the planar structure of graphene were taken from Gray *et al.*^35^ The x- and y-dimensions of the graphene sheet were set to 100 Å and 50 Å, respectively. A total of two flCNT sheets (four graphene layers) were modelled, accounting for a total of 7872 atoms. Fig. 1 shows the two models having 0° and 90° orientations between the two flCNT sheets.

### 2.2 Crosslinking

**Fig. 2:**
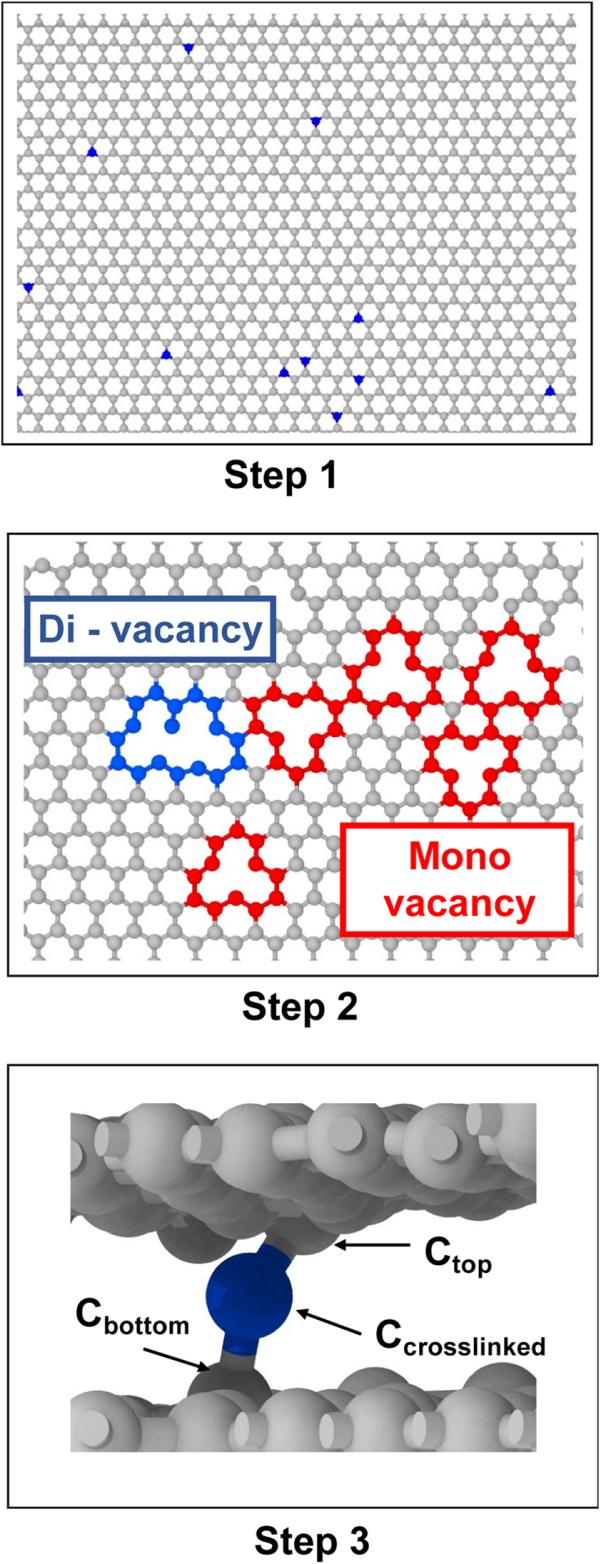
Steps for building crosslinked models: Step 1 – random selection of carbon atoms (blue color), Step 2 – displacement of selected atoms from their initial position resulting in di-vacancy (blue color) and mono vacancy formation (red color), Step 3 – the displaced carbon atom forms a crosslink between the two graphene sheets (blue color).

The interlayer spacing between graphene sheets is about 3.44 Å,^36^ while the length of a single C–C bond is 1.55 Å.^37^ Therefore, it is not possible to form a single stable C–C bond between the two graphene layers. Thus, an in-house python script was used to induce the crosslinking within and between the graphene sheets. The following procedure was followed (each step is shown graphically in Fig. 2):

• Step 1: a carbon atom on the graphene sheet was randomly chosen.

• Step 2: the selected carbon atom was displaced from its initial position towards the neighbouring sheet, which resulted in mono-valency or di-valency defects. This displaced atom is henceforth referred to as the crosslinked atom (C_crosslinked_).

• Step 3: the carbon atom (C_top_) above and attached to C_crosslinked_ was displaced in the downward direction by 0.5 Å, and the carbon atom (C_bottom_) below and attached to C_crosslinked_ was displaced upwards by 0.3 Å, which resulted in the formation of a covalent bond chain between the two graphene sheets.

• Step 4: this process was repeated for each model until the desired crosslink density was achieved.

After the formation of crosslinks, the models were equilibrated at room temperature (300 K) at 1 atm pressure using the fixed pressure and temperature (NPT) ensemble. The equilibration simulation was run for 500 picoseconds (ps) with a timestep of 1 femtosecond (fs). For both orientation cases, five crosslinked models having 0, 1, 3, 5, 10 and 20% were created. A total of five independent models were created for each orientation case and crosslink density to account for statistical deviations in the predicted properties. Representative images of these models are included in the ESI (Fig. S1 and S2†). The evolution of the chemical bonding of the flCNTs with increasing crosslinking is shown in Fig. S3.†

**Fig. 3:**
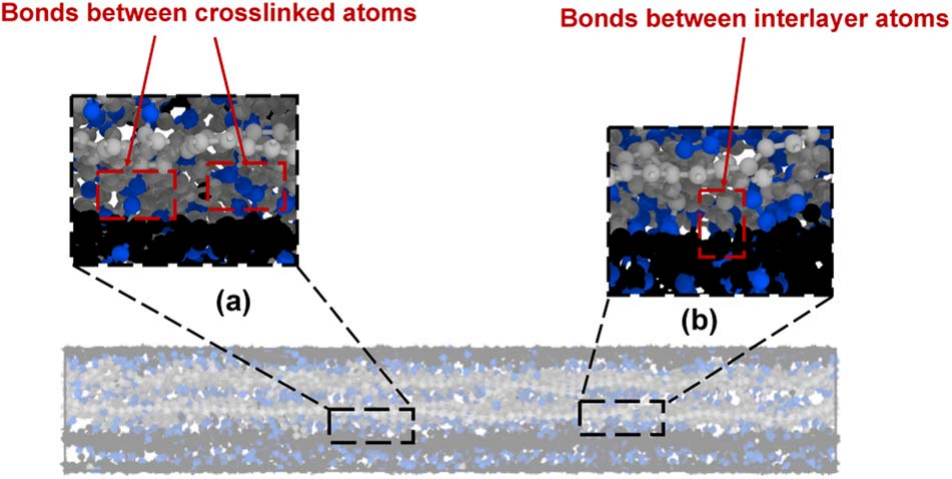
Bonds between (a) crosslinked atoms and (b) interlayer atoms. The blue-colored atoms are the crosslink atoms, while the grey- and black-colored atoms are the flCNT atoms.

**Fig. 4:**
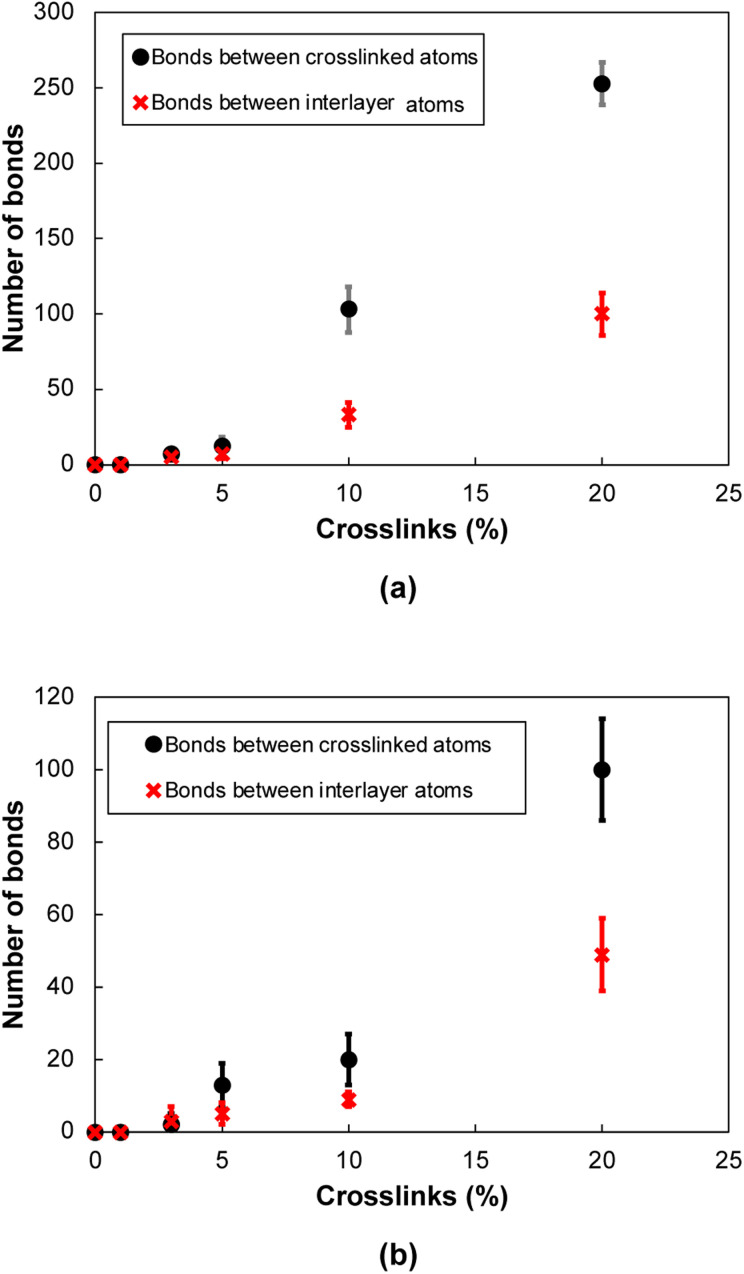
Number of bonds as a function of crosslinks for (a) 0° orientation, (b) 90° orientation. The uncertainty represents the standard deviation between the independently-built replicate models.

In general, two different types of covalent bond formations occur during the crosslinking process. Bonds can form between crosslinking atoms and the flCNTs (Fig. 3a), and between carbon atoms in a single flCNT layer after a local disruption of the aromatic structure with crosslinking (Fig. 3b). Fig. 4a and b show the number of crosslinked and interlayer bonds formed in the 0° and 90° orientations, respectively. The data in the figures demonstrate that the bonding between crosslinked atoms was much higher than the interlayer bonding during the crosslinking simulations for both 0° and 90° orientations.

### 2.3 Shear simulation

Previous MD studies utilized pull-out simulations to predict interfacial shear strength.^18–21,38–40^ Pull-out simulations were performed herein on each replicate of the crosslinked models for each orientation case to investigate the interfacial shear strength between the two flCNT sheets. The interfacial shear strength was predicted by applying a pulling force on each carbon atom on one flCNT sheet (using the “fix addforce” LAMMPS command), while the movement of the other flCNT sheet was restrained using a spring of stiffness 100 kcal mol^−1^ Å^−2^/Å (using the “fix spring” LAMMPS command). The pulling force was incrementally increased every 0.1 fs by 2 × 10^−6^ kcal mol^−1^ Å^−1^. The pull-out simulations were carried out at 300 K and 1 atm using the NPT ensemble for 1 nanosecond (ns).

### 2.4 Transverse tension

To predict the transverse strength between the two flCNT sheets, the simulation box was uniaxially deformed in tension along the *z*-direction, which is the direction transverse to flCNT surface. The procedure is similar to those used for predicting interfacial transverse strength in flCNT/polymer composites.^18–20^ These simulations were performed on each replicate of the crosslinked models for both orientation cases at room temperature (300 K) and 1 atm pressure using the NPT ensemble. The simulation box was deformed at a rate of 2 × 10^8^ s^−1^ and a 200% total strain was applied to capture the complete failure of the interface. The stress–strain response along the *z*-direction was recorded over the entire strain range. Three metrics were determined from the stress–strain curve. First, the stiffness was determined from the slope of stress/strain response in the initial linear region. Second, the ultimate strength was determined from the maximum stress value achieved during the simulation. Third, the toughness was calculated from the total area under the stress–strain curve.

### 2.5 Pulling along armchair and zigzag direction

To predict the effect of the crosslinking on the axial properties of flCNTs, the simulation boxes were uniaxially deformed in the armchair and zigzag directions of the graphene sheets (Fig. S5†). These simulations were performed at room temperature (300 K) and pressure (1 atm) using the NPT ensemble. The simulation boxes were deformed at a rate of 2 × 10^8^ s^−1^ with a total applied strain of 100% to fully capture the failure of each model. These simulations were performed on each replicate of the crosslinked models for the 0° orientation case. For each simulation, the stiffness, ultimate strength, and toughness were determined.

## 3 Results

The results of the interfacial shear strength, transverse tension, and axial tension simulations are discussed in this section.

### 3.1 Interfacial shear strength

**Fig. 5:**
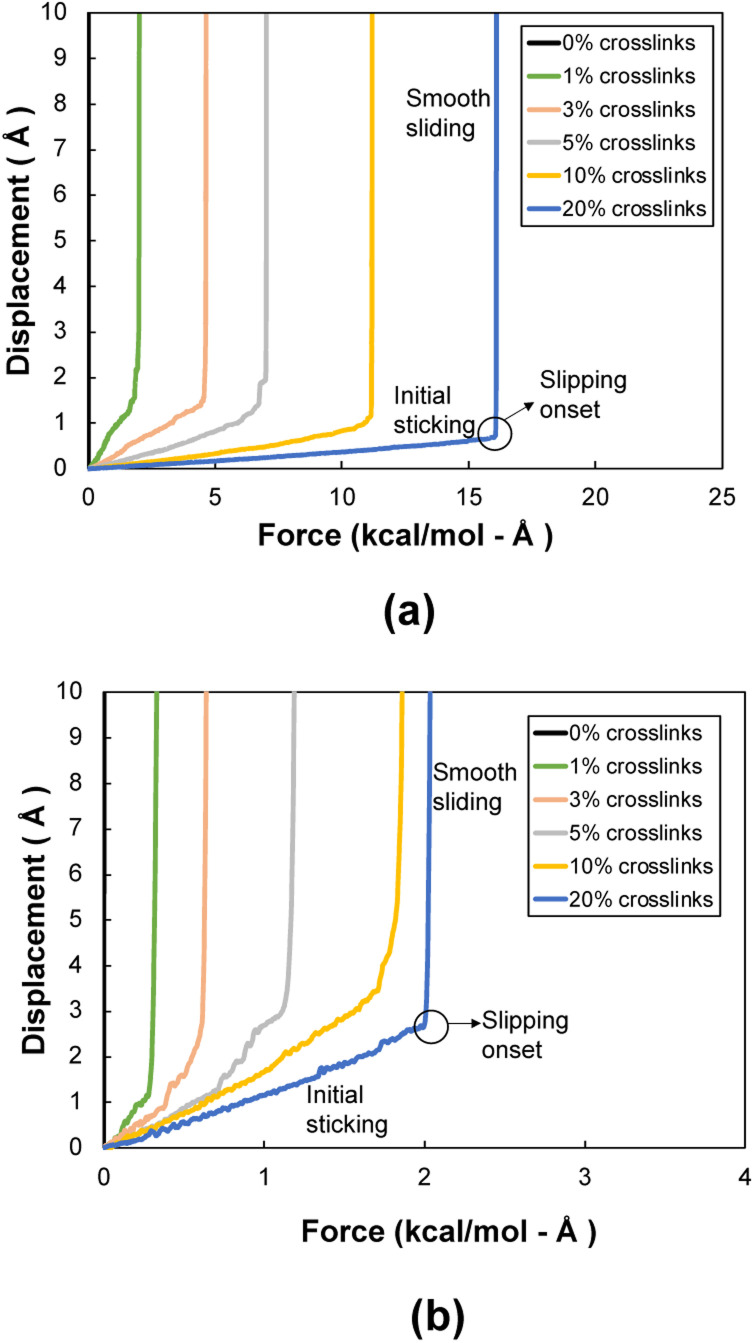
Representative displacement *vs.* force profiles of (a) 0° and (b) 90° orientation models.

Fig. 5a shows a representative flCNT shear displacement *vs.* pull-out force plot for the 0° orientation for each level of crosslinking. Three distinct regions are observed in the displacement profile: initial sticking, slipping onset, and smooth sliding. The sticking region corresponds to the resistance to shearing caused by long-range van der Waals forces and flCNT crosslinks. The onset of slipping corresponds to the scission of the crosslinks, each of which occurs in quick succession after the failure of the first crosslink. In the smooth sliding region, only the van der Waals forces of adjacent flCNTs act on the atoms, thus there is little resistance to pull-out of the flCNT. Snapshots of a representative 0° orientation system having 1% crosslinks undergoing pull-out is included in the ESI (Fig. S4†).

Fig. 5b shows a representative flCNT displacement *vs.* pull-out force plot for the 90° orientation for each level of crosslinking. A similar behavior is observed in these systems as with the 0° systems, except the force corresponding to the slipping onset is much lower because the overlapping surface area in the 90° orientation is lower than that in the 0° orientation, which results in a lower number of crosslinks between the two flCNT sheets.

### 3.2 Transverse tension

**Fig. 6:**
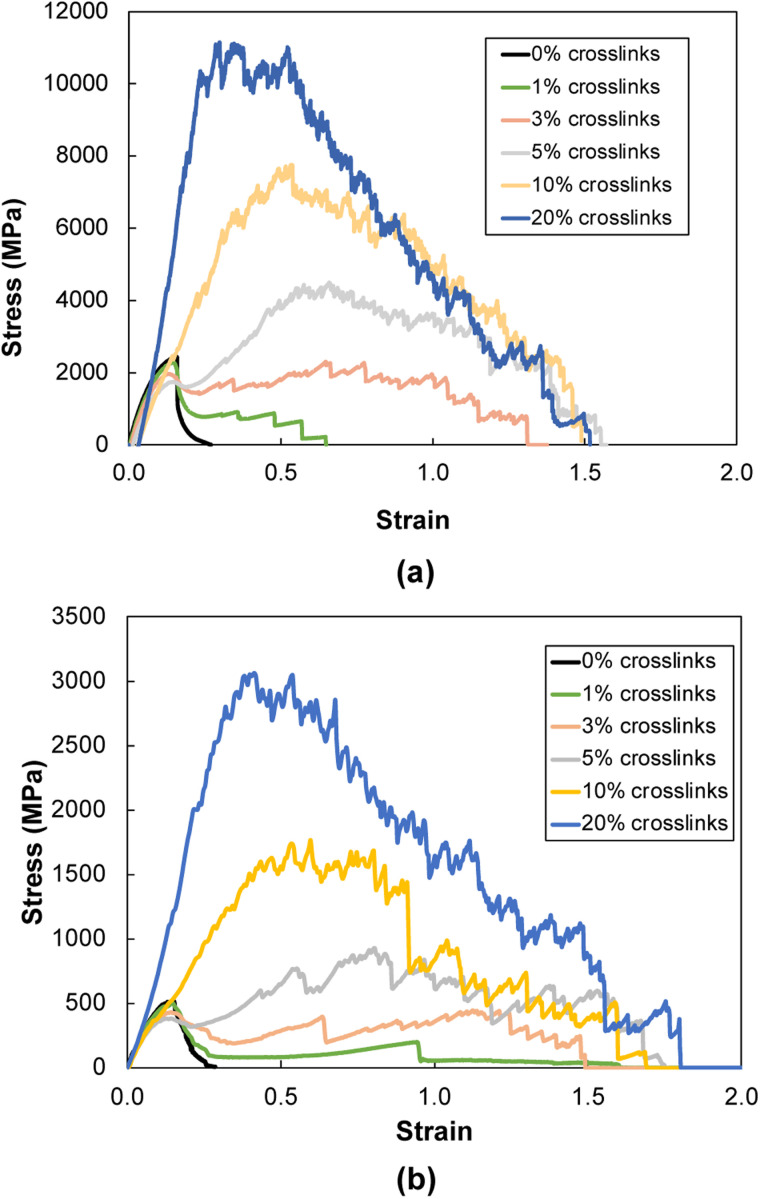
Representative stress–strain plots during transverse tension for (a) 0° and (b) 90° orientation models.

**Fig. 7:**
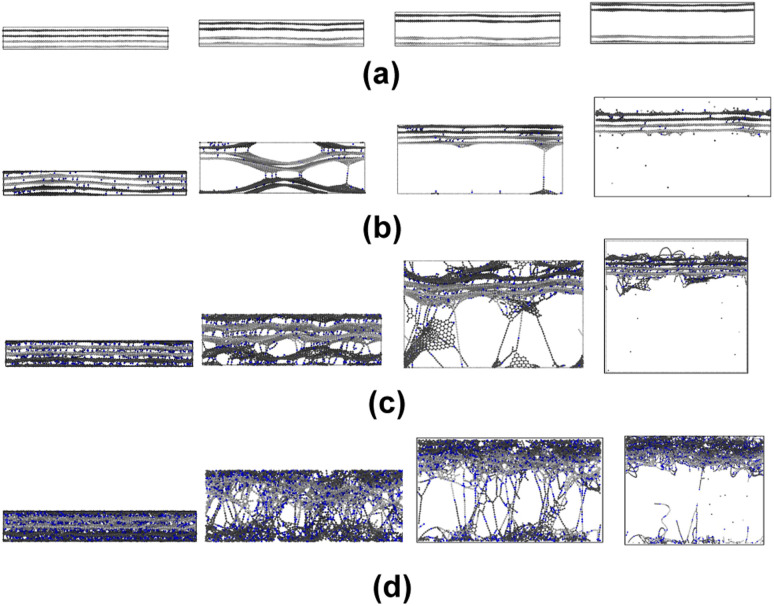
Snapshots of a representative 0° orientation model with various degrees of crosslinking undergoing transverse tension for (a) 0% crosslinking, (b) 1% crosslinking, (c) 5% crosslinking, (d) 20% crosslinking.

**Fig. 8:**
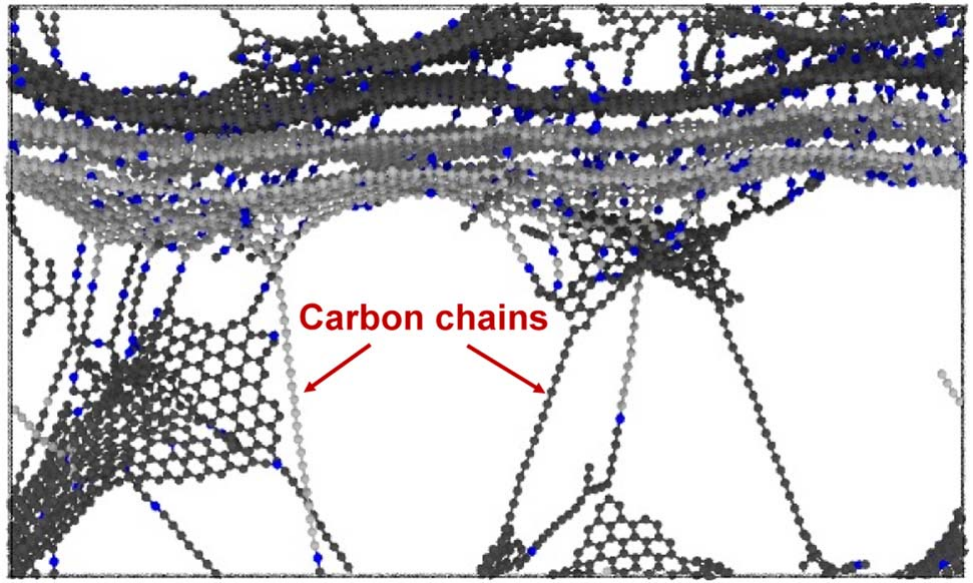
Snapshot of carbon chain formation while undergoing transverse tension.

Fig. 6a and b show representative stress–strain curves for transverse tension for the 0° and 90° orientation models, respectively. A significant increase in the toughness of the interfacial regions is observed for increasing levels of crosslinking. Snapshots of a representative 0° orientation model having 0% crosslinks between and within the flCNT sheets are shown in Fig. 7a. Fig. 7a shows that as the model is strained in the transverse direction, failure is observed in the region between the two flCNT sheets which is dominated by van der Waals interactions. Snapshots of representative 0° orientation models having 1%, 5%, 20% crosslinks between and within the flCNT sheets are shown in Fig. 7b–d, respectively. As the crosslinked models are strained in the transverse direction, the formation of sp-hybridized carbon atom chains (Fig. 8) is observed, and the concentration of carbyne chains increases with increases in the number of crosslinks. Carbyne chains are relatively reactive and unstable,^41,42^but can exist on the short time scales of MD simulations.^43^

**Fig. 9:**
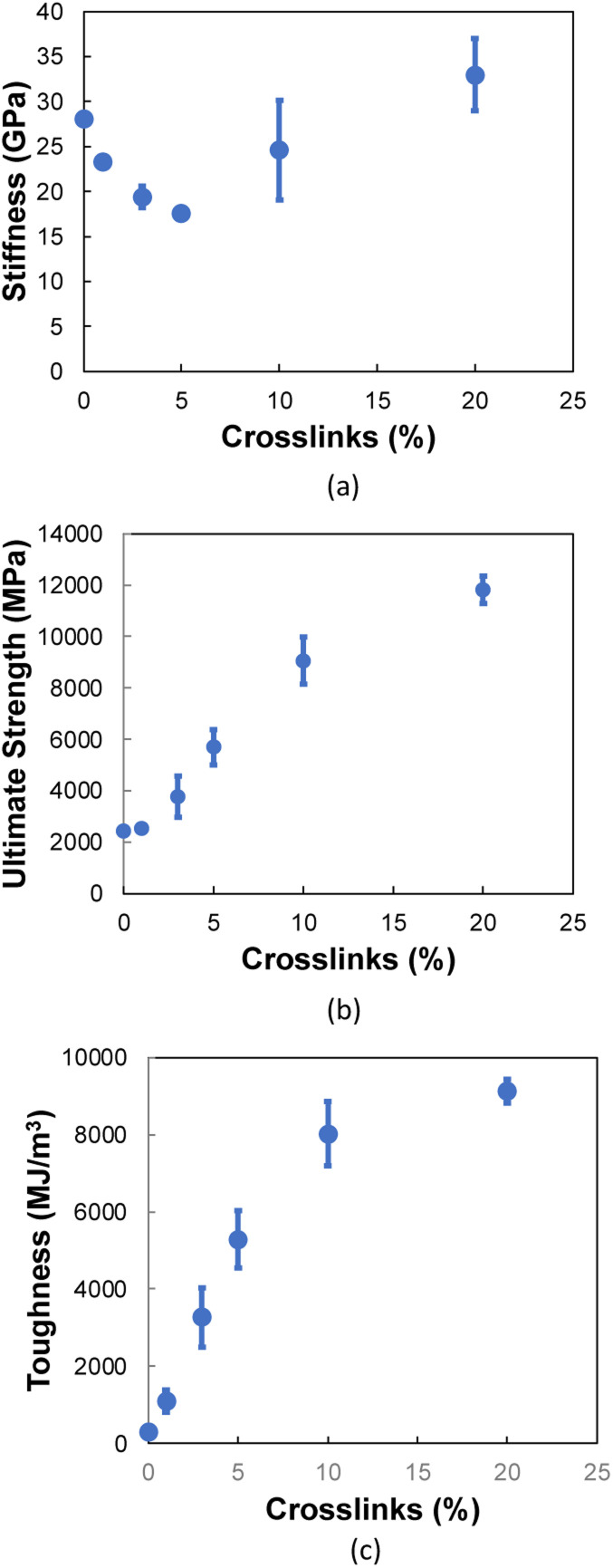
Mechanical properties as a function of crosslinks for the 0° orientation in terms of (a) stiffness, (b) ultimate strength, (c) toughness. Each data point represents the average of five MD replicates and the vertical error bars represent the standard deviation.

Fig. 9a shows the stiffness as a function of crosslinking for the 0° orientation. The addition of crosslinks increases the defects in the aromatic structure, which initially disrupts the integrity of the flCNT sheets, resulting in a decrease in stiffness up to 5% crosslinking. However, after 5% crosslinking, a sharp increase in stiffness is observed, which is attributed to the increasing number of covalent bonds between the flCNT sheets with increasing crosslinking.

For the ultimate strength (Fig. 9b) and toughness (Fig. 9c) of the 0° orientation, an increase is observed immediately after the addition of crosslinks between and within the two flCNT sheets. This increase is attributed to the increasing levels of interfacial load transfer with the addition of chemical crosslinks.

**Fig. 10:**
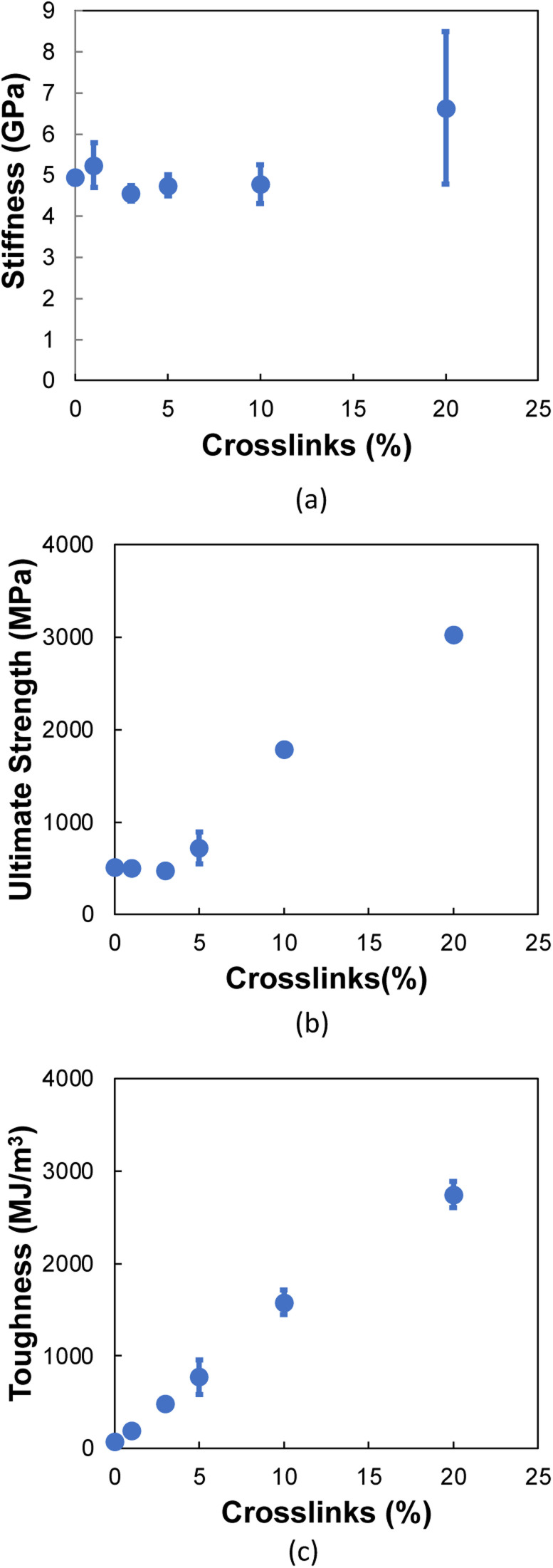
Mechanical properties as a function of crosslinks for the 90° orientation in terms of (a) stiffness, (b) ultimate strength, (c) toughness. Each data point represents the average of five MD replicates and the vertical error bars represent the standard deviation.

Fig. 10a shows the stiffness as a function of crosslinks for the 90° orientation. The stiffness values remain nearly constant up to 10% crosslinking, followed by an increase at 20% crosslinking. For ultimate strength (Fig. 10b), a near constant trend is observed up to 5% crosslinking, where a significant increase is observed for increasing crosslinking levels. Fig. 10c shows a linear increase in the toughness as a function of crosslinking, which is due to the formation of carbon chains (Fig. 8) formed during transverse tension, which allows a load-bearing feature that persists until failure, resulting in higher energy absorption.

### 3.3 Tension along armchair and zigzag directions

**Fig. 11:**
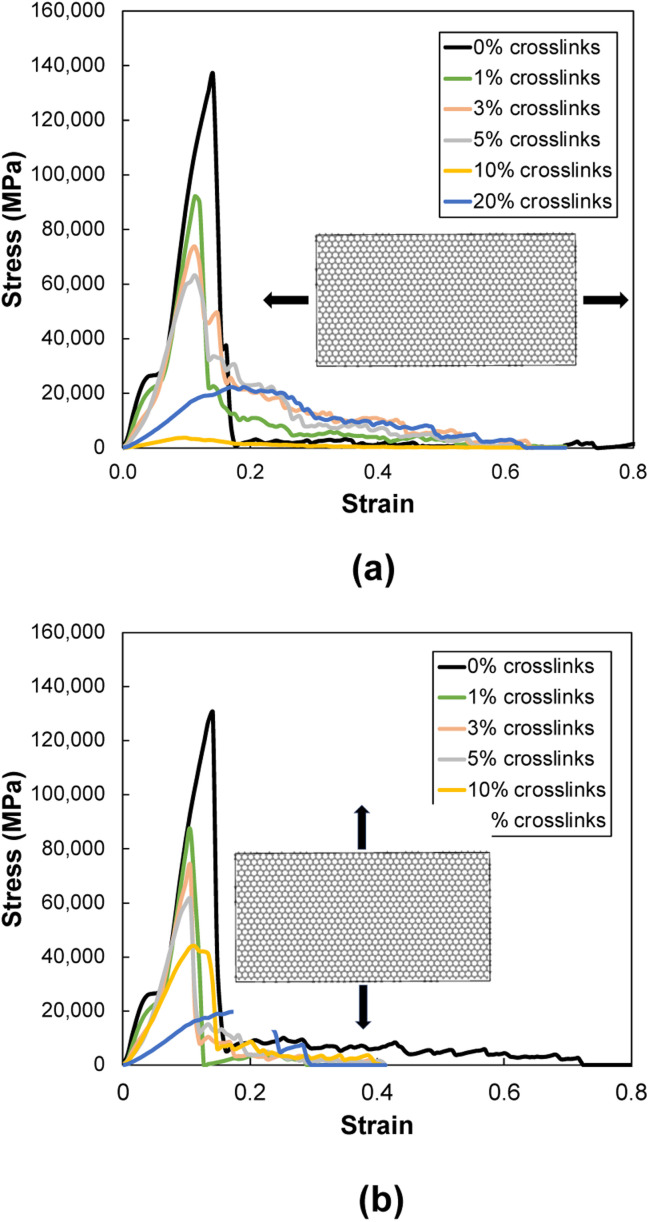
Representative stress–strain plots during tension loading along the (a) armchair and (b) zigzag directions.

**Fig. 12:**
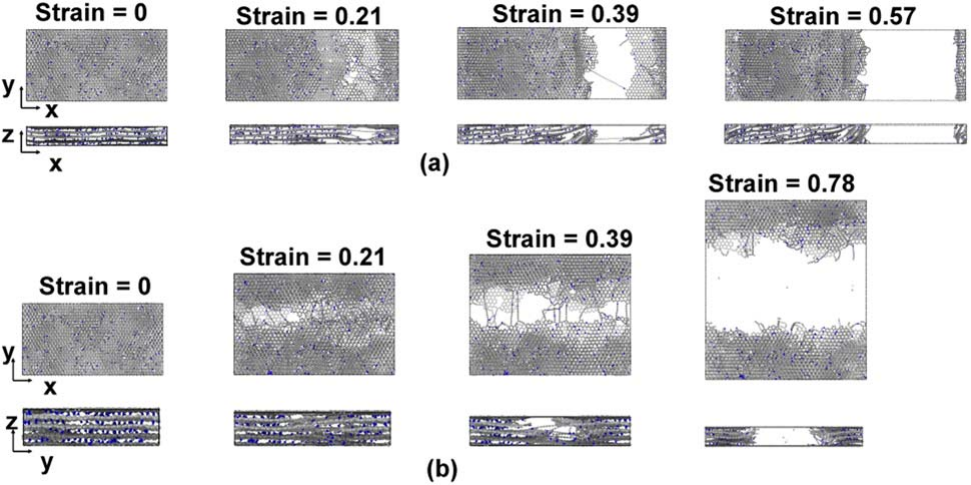
Snapshots of the 3% crosslinked model undergoing tension along the (a) armchair and (b) zigzag directions.

Fig. 11a and b show representative stress–strain curves for tension loading along the armchair and zigzag directions, respectively. Unlike the transverse tension behavior, decreases in the mechanical performance with increases in crosslinking are observed when the flCNT sheets were pulled along both armchair and zigzag directions. Snapshots of the 3% crosslinked model strained along the armchair and zigzag directions are shown in Fig. 12a and b, respectively. As the simulation box is pulled along the armchair and zigzag directions, the formation of carbon atom chains is observed. The carbon chains are formed between segments of the same sheet and different sheets.

**Fig. 13:**
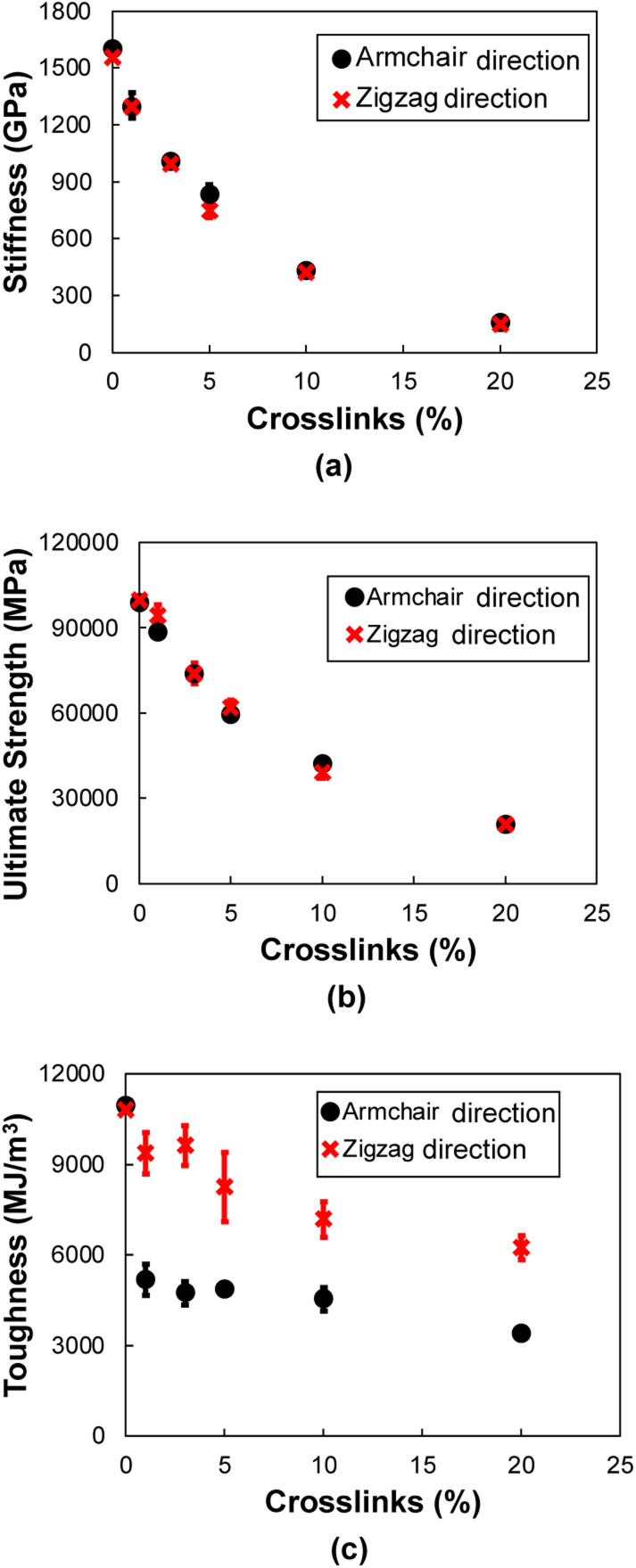
Mechanical properties as a function of crosslinking for tensile deformation in armchair and zigzag directions for (a) stiffness, (b) ultimate strength, (c) toughness. Each data point represents the average of five MD replicates and the vertical error bars represent standard deviation.

Fig. 13a–c show the stiffness, ultimate strength, and toughness, respectively, as a function of crosslinking for flCNT sheets subjected to tensile loading along the armchair and zigzag directions. A decrease in all three properties is observed for increasing crosslinking. As the amount of crosslinking increases, the damage within the flCNT sheets also increases. Because the initiation point of failure is located at a crosslinking site (Fig. S6†), the failure of the structure occurs at a much lower strain value relative to the model having 0% crosslinking. From Fig. 13c it is evident that higher values of toughness are observed when the sheets are pulled along the zigzag direction. This is because of the higher number of carbon chains formed during extended tensile deformation along the zigzag direction (Fig. 12), which requires more applied strain energy for failure.

## 4 Conclusions

The results of this study clearly show that irradiation-induced crosslinking has a significant impact on the mechanical performance of flCNT junctions in CNT yarn materials. For both 0° and 90° flCNT orientations subjected to transverse tension, increasing levels of crosslinking result in increasing amounts of flCNT interfacial toughness, stiffness, and strength. The formation of covalent bonds between flCNTs during the irradiation process provides for effective interfacial load transfer. Once interfacial failure initiates, the primary toughening mechanisms for the crosslinked systems is the formation of sp-hybridized carbon chains that continue to carry load until final interfacial rupture.

For tension applied along armchair and zigzag directions of flCNTs, increasing levels of crosslinking result in decreasing values of modulus, strength, and toughness. These decreases are a result of the damage that is induced in the aromatic structure of the flCNTs when crosslinked. Similar to the behavior in transverse tension, the primary mechanisms that provides toughness is the formation of carbon chains along the direction of applied tension, especially along the zigzag direction.

Based on these results, it is clear that irradiation-induced crosslinking is beneficial in composite systems in which interfacial load transfer between flCNTs is of primary importance. This is especially relevant for CNT yarns, which contain substantial levels of flCNTs which can easily slide past each other with localized shear forces. If increased levels of shearing resistance between flCNTs can be induced, then improved load transfer is expected, which can improve the overall performance of CNT yarns, even though the individual flCNTs may lose some mechanical integrity with crosslinking. It is expected that these conclusions could also apply to other types of CNT-based and graphene-based composites, as the chemical structure of the carbon is similar in these aromatic systems.

## Author contributions

PSG: conceptualization, data curation, investigation, methodology, visualization, writing – original draft, writing – review & editing; MK: investigation, methodology, writing – review & editing; AD: project administration, writing – review & editing; GMO: funding acquisition, investigation, methodology, resources, supervision, project administration, writing – review & editing.

## Conflicts of interest

There are no conflicts of interests.

## Supplementary Material

RA-012-D2RA05550C-s001
