# Supplementary material for: Computational study of effect of radiation induced crosslinking on the properties of flattened carbon nanotubes
Source: RSC Adv. 2022 Oct 11;12(45):28945–53. doi: 10.1039/d2ra05550c (PMC9552316; doi:10.1039/d2ra05550c)
Supplement: RA-012-D2RA05550C-s001 [file RA-012-D2RA05550C-s001.pdf]

Electronic Supplementary Material (ESI) for RSC Advances.
This journal is © The Royal Society of Chemistry 2022

Supporting Information
for

Computational study of Effect of Radiation Induced Crosslinking on the Properties of Flattened Carbon Nanotubes

Prashik S. Gaikwad[1], Malgorzata Kowalik[2], Adri van Duin[2], Gregory M. Odegard[1*]

[1]Michigan Technological University, Houghton, MI 49931, USA.
[2]Pennsylvania State University, State College, PA 16801, USA.

*Corresponding author: gmodegar@mtu.edu

0° orientation crosslinked models

Figure S1 shows snapshots of representative MD models of relaxed crosslinked flCNTs having a 0° orientation with increasing levels of crosslinking.

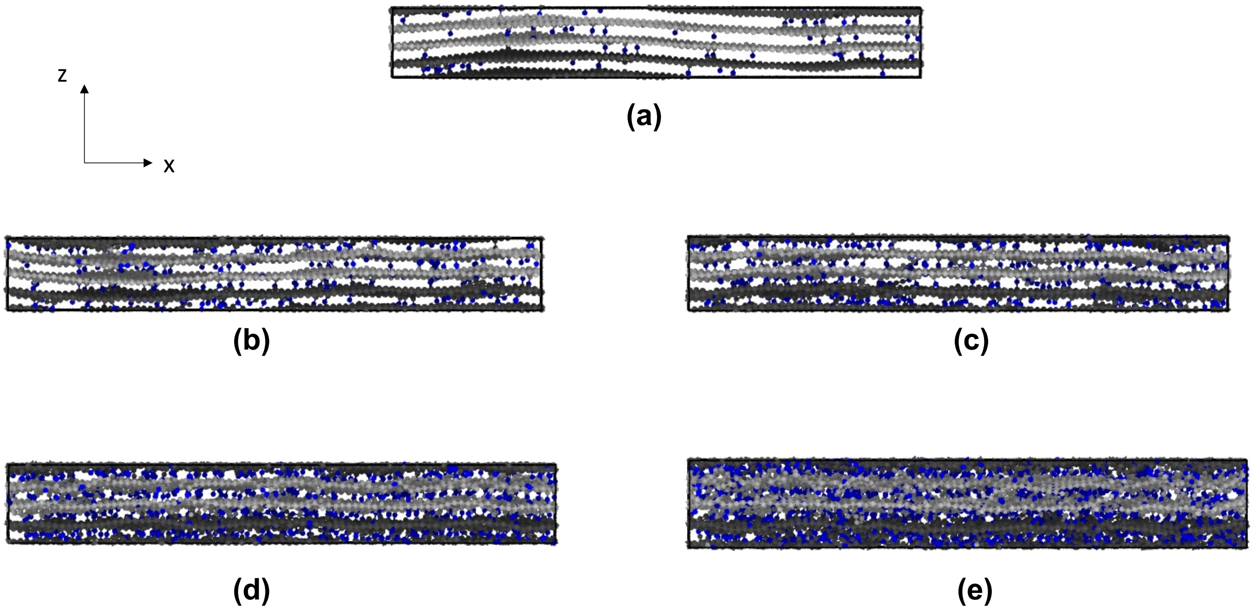

Figure S1: Images of relaxed crosslinked structures having a 0° orientation with (a) 1% crosslinks, (b) 3% crosslinks, (c) 5% crosslinks, (d) 10% crosslinks, (e) 20% crosslinks.

90° orientation crosslinked models

Figure S2 shows snapshots of representative MD models of relaxed crosslinked flCNTs having a 90° orientation with increasing levels of crosslinking.

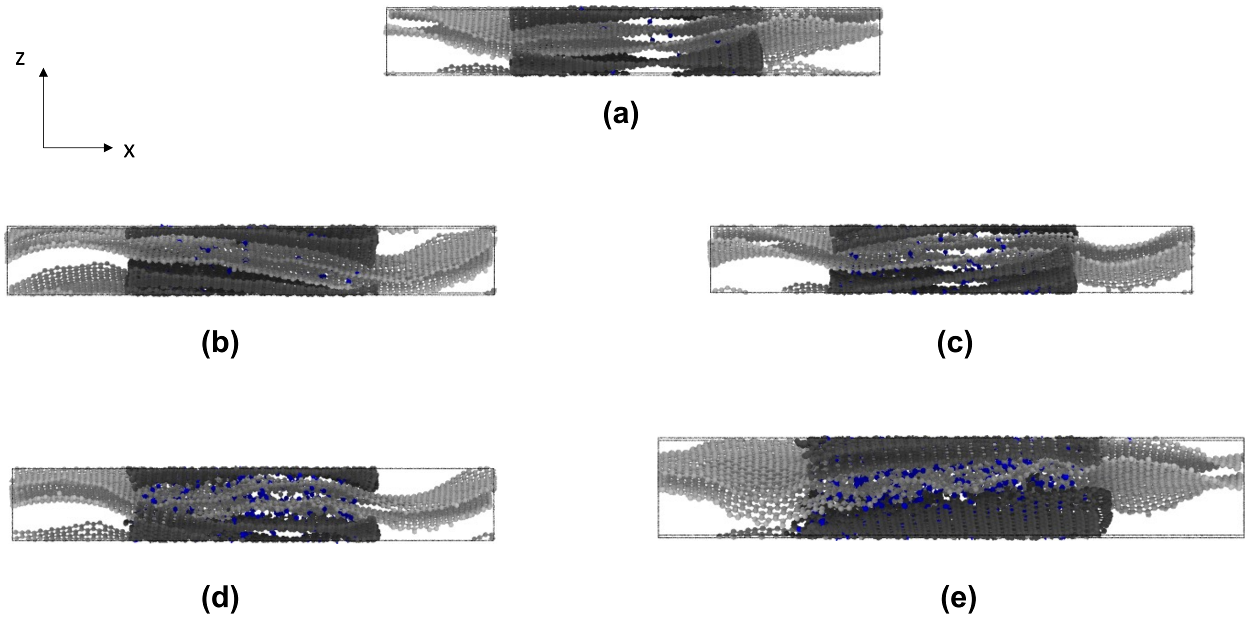

Figure S2: Images of relaxed crosslinked structures having a 90° orientation with (a) 1% crosslinks, (b) 3% crosslinks, (c) 5% crosslinks, (d) 10% crosslinks, (e) 20% crosslinks.

Evolution of bonding as function of crosslinking

Figures S3a and S3b show the evolution of the chemical bonding of the flCNT sheets for increasing levels of crosslinking for the 0° and 90° orientations, respectively. For the 0% crosslinked system, content of 6-membered rings and sp^2 hybridization are 100% (aromatic carbon atoms), as the structure is pristine (without any defects). As the level of crosslinking increases, a decline in the relative content of 6-membered rings and sp^2 hybridization is observed. This decline is due to the increasing disruption of the aromatic structure with increasing crosslinking.

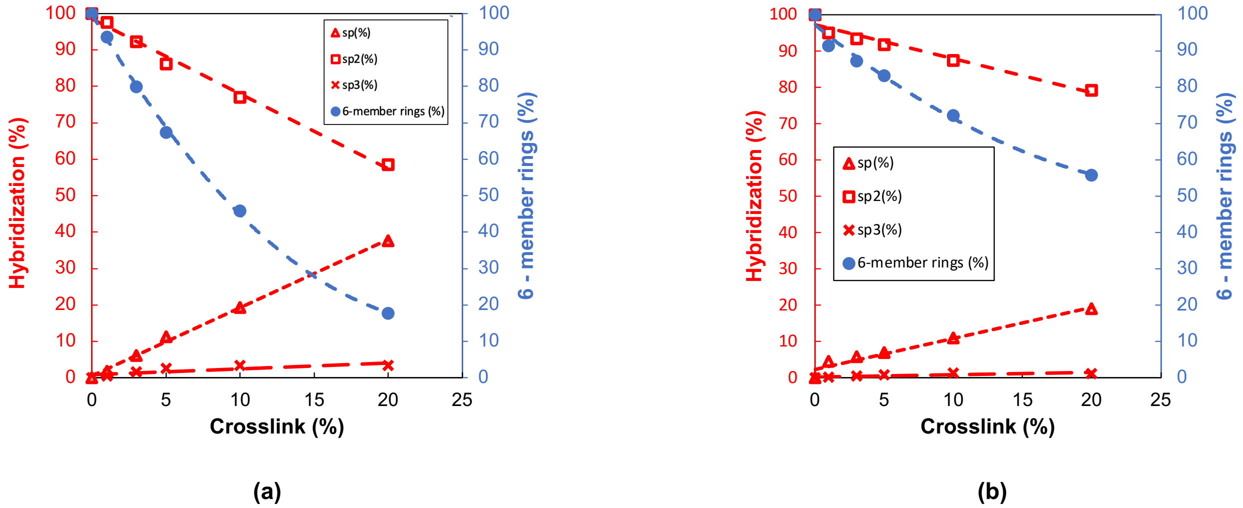

Figure S3: Evolution of flCNT model as a function of crosslinks for (a) 0° orientation, (b) 90° orientation.

Pull-out Simulations

Figure S4a shows a representative initial structure before any pull-out force is applied on the flCNT sheet. When the pull-out force is incrementally increased, the crosslinks are stretched in the direction of pull (Figure S4b). The breaking of all crosslinks is observed at a pull-force of 2.046 kcal/mol- Å for the model having 1% crosslinks within and between the two flCNT sheets (Figure S4c).

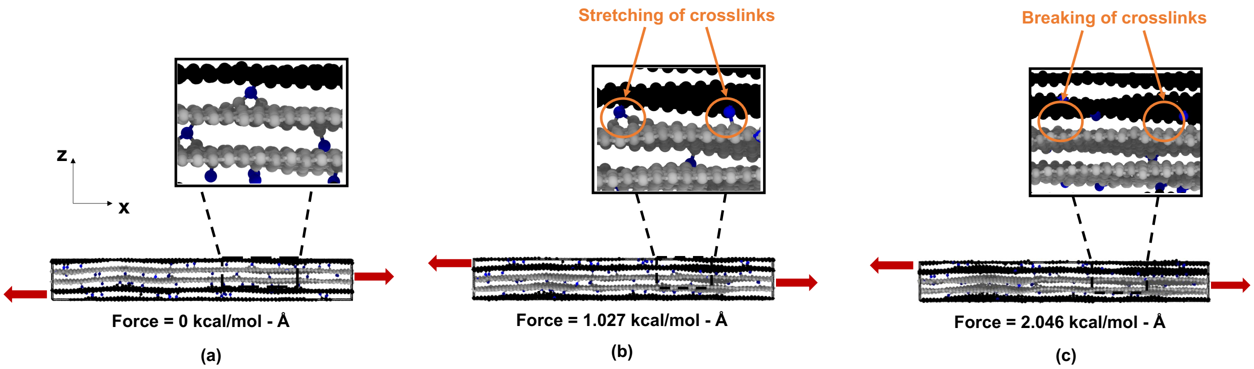

Figure S4: Snapshots of the 0° orientation model having 1% crosslinks undergoing a simulated pull-out of the flCNTs. (a) Pull-out force = 0 kcal/mol-Å, (b) Pull-out force = 1.027 kcal/mol-Å, (c) Pull-out force = 2.046 kcal/mol-Å.

Pulling along armchair and Zigzag direction

Figure S5 shows the armchair and zigzag directions of the flCNT sheets. Figures S6a and S6b show the initiation and propagation of failure while pulling the flCNT sheets along the armchair and zigzag directions, respectively for the 3% crosslinked model. The failure initiates locally at a

cluster of crosslinking sites, and propagates by connecting the defect sites along its path, which results in the total failure of the structure.

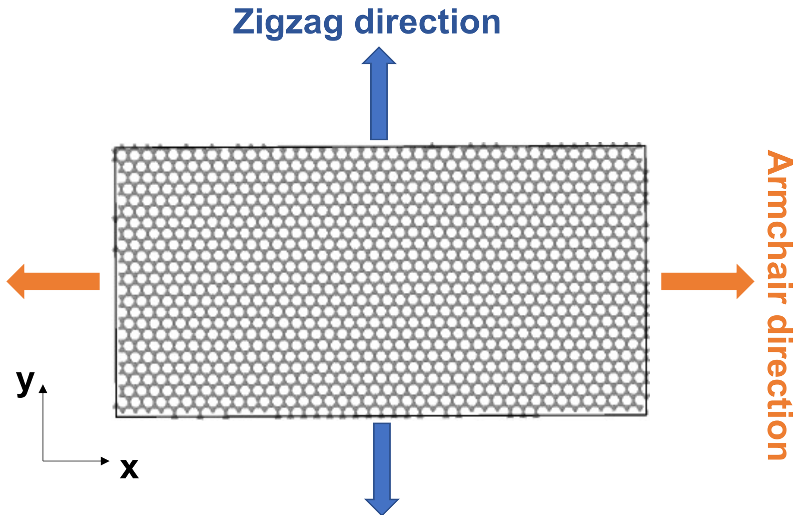

Figure S5: Armchair and zigzag directions of flCNT sheets.

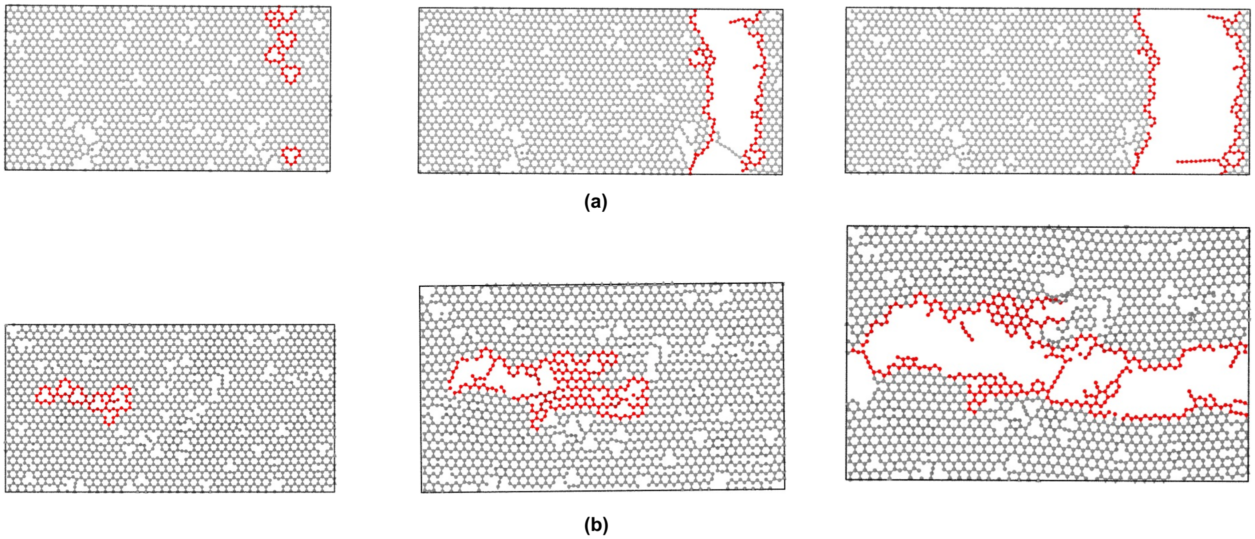

Figure S6 : Snapshots of crack initiation and propagation for the 3% crosslinked model during applied tension along the (a) armchair and (b) zigzag directions. The red-colored atoms highlight the defect sites from where the crack initiates and propagates. Three layers of graphene sheets are deleted for visual clarity.
